# Cyanobacterial neurotoxin BMAA and brain pathology in stranded dolphins

**DOI:** 10.1371/journal.pone.0213346

**Published:** 2019-03-20

**Authors:** David A. Davis, Kiyo Mondo, Erica Stern, Ama K. Annor, Susan J. Murch, Thomas M. Coyne, Larry E. Brand, Misty E. Niemeyer, Sarah Sharp, Walter G. Bradley, Paul Alan Cox, Deborah C. Mash

**Affiliations:** 1 Department of Neurology, Miller School of Medicine, University of Miami, Miami, Florida, United States of America; 2 Department of Chemistry, University of British Columbia, Kelowna, British Columbia, Canada; 3 Office of the District 21 Medical Examiner, Fort Myers, Florida, United States of America; 4 Divisions of Marine Biology and Fisheries and NSF/NIEHS Oceans and Human Health Center, Rosenstiel School of Marine and Atmospheric Science, University of Miami, Miami, Florida, United States of America; 5 Marine Mammal Rescue and Research, International Fund for Animal Welfare (IFAW), Yarmouth Port, Massachusetts, United States of America; 6 Brain Chemistry Labs, Institute for Ethnomedicine, Jackson Hole, Wyoming, United States of America; 7 Department of Molecular and Cellular Pharmacology, Miller School of Medicine, University of Miami, Miami, Florida, United States of America; Universidad de los Andes, COLOMBIA

## Abstract

Dolphin stranding events occur frequently in Florida and Massachusetts. Dolphins are an excellent sentinel species for toxin exposures in the marine environment. In this report we examine whether cyanobacterial neurotoxin, β-methylamino-L-alanine (BMAA), is present in stranded dolphins. BMAA has been shown to bioaccumulate in the marine food web, including in the muscles and fins of sharks. Dietary exposure to BMAA is associated with the occurrence of neurofibrillary tangles and β-amyloid plaques in nonhuman primates. The findings of protein-bound BMAA in brain tissues from patients with Alzheimer’s disease has advanced the hypothesis that BMAA may be linked to dementia. Since dolphins are apex predators and consume prey containing high amounts of BMAA, we examined necropsy specimens to determine if dietary and environmental exposures may result in the accumulation of BMAA in the brains of dolphins. To test this hypothesis, we measured BMAA in a series of brains collected from dolphins stranded in Florida and Massachusetts using two orthogonal analytical methods: 1) high performance liquid chromatography, and 2) ultra-performance liquid chromatography with tandem mass spectrometry. We detected high levels of BMAA (20–748 μg/g) in the brains of 13 of 14 dolphins. To correlate neuropathological changes with toxin exposure, gross and microscopic examinations were performed on cortical brain regions responsible for acoustico-motor navigation. We observed increased numbers of β-amyloid^+^ plaques and dystrophic neurites in the auditory cortex compared to the visual cortex and brainstem. The presence of BMAA and neuropathological changes in the stranded dolphin brain may help to further our understanding of cyanotoxin exposure and its potential impact on human health.

## Introduction

Harmful algal blooms (HABs) are becoming increasingly frequent in fresh water lakes, estuaries, and the sea [1–3] likely due to climate warming and increased phosphorous and nitrogen effluents [2]. HABs are sometimes associated with widespread dying-off of sea grass and fish ascribed to the large biomass of algae producing hypoxic conditions in the water [4]. HABs composed of cyanobacteria contain multiple cyanotoxins including microcystins, nodularin, cylindrospermopsin, and anatoxins, while those composed of dinoflagellates can contain brevitoxins [5–7]. Acute and chronic exposures to such toxins can be harmful to both humans and animals resulting in respiratory illnesses, severe dermatitis, mucosal damage, cancer, organ failure and death [7].

The cyanotoxin, β-*N*-methylamino-*L*-alanine (BMAA), has been linked to several neurodegenerative diseases [8–11]. BMAA has been shown to pass the blood-brain barrier where it is incorporated into brain proteins [3,12–14] inducing misfolding and aggregation [13]. Chronic dietary exposure to BMAA has been shown to trigger neurodegenerative changes in experimental models including non-human primates [15–17]. BMAA has also been detected in postmortem brain tissues of patients with Amyotrophic Lateral Sclerosis (ALS), Parkinsonism Dementia Complex of Guam (ALS/PDC) and Alzheimer’s disease (AD) [18–20]. Epidemiological studies support a link between BMAA and neurodegenerative disease, since people living in close proximity of lakes with frequent cyanobacteria blooms have increased an incidence of ALS [21–25]. BMAA has been shown to accumulate in long-lived apex predators like sharks [26], as well as throughout the South Florida marine food web [27,28].

Dolphins that inhabit Florida coastal waters are often exposed to recurring HABs [29–31]. Coastal subpopulations of bottlenose dolphins in the southeastern United States have an increased risked of cyanobacteria bloom exposure, compared to common dolphins, a pelagic species, which spend relatively little time in coastal or near-shore environments [27,32]. Bottlenose dolphins have a median lifespan of 30 to 32 years with an maximum of 50 years in captivity [33]. Irrespective of age or sex, bottlenose dolphins have been observed stranded all along the coastal United States [34–36]. Stranding events have been attributed to trauma [37], infections [35], neuropathology [38], navy sonar [39,40] or toxin exposures [29]. Regardless of cause of stranding, dolphins are valuable bio-indicators of the health of the marine environment and provide clues of potential environmental risks to humans. We hypothesize that dolphins are likely to accumulate BMAA both by exposure to HABs and by the ingestion of prey previously exposed to the cyanotoxin [27,28].

In this study, we examined an opportunistic sample of brain tissues from fourteen stranded dolphins obtained from the Rosenstiel School of Marine and Atmospheric Science and the International Fund for Animal Welfare (IFAW). BMAA was measured in select brain regions of stranded dolphins. In addition, we examined brain tissues of seven dolphins stranded in Massachusetts to seek a correlation between neuropathological changes and the presence of BMAA.

## Material and methods

### Dolphins

Adult (n = 9) and sub-adult (n = 5) stranded dolphins were obtained from ten different sites in Florida and Massachusetts between 2005 and 2012 (S1 Table). The seven Florida dolphins were found stranded in sites known to have recurring harmful algal blooms: Atlantic Ocean [41], Banana River [42], Indian River Lagoon [42,43] and Gulf of Mexico [30]. The seven Massachusetts dolphins were found stranded in various sites in Cape Cod Bay [44]. Cyanobacteria species capable of producing BMAA were detected in all these regions as determined by surveillance data from the Phytoplankton Monitoring Network (S2 Table) [45]. Dolphins were classified as adult or sub-adult based on body length from the Geraci & Lounsbury 1993 stranding guide [46]. Dolphins varied in manner of stranding, species and sex (S1 Table). Six of fourteen dolphins (43%) were observed alive while stranded. Physical assessments were performed on the dolphins when first sighted [46]; those assessed to be in poor health and unable to be released were euthanized on site, followed by a full necropsy within 24 to 48 hours by IFAW. Dolphin specimens in Massachusetts were obtained under a Stranding Agreement between National Ocean and Atmospheric Administration (NOAA) National Marine Fisheries Services Greater Atlantic Region Fisheries Office and IFAW. That agreement enables access to and management of stranded pinnipeds and cetaceans, and where applicable, landowners were consulted when animals were located on private property. Clinical assessments and euthanasia were conducted according to IFAW’s protocols approved by the Woods Hole Oceanographic Institution Institutional Animal Care and Use Committee. Euthanasia was done by intravenous barbiturate, following guidelines from Barco *et al* 2016 [47]. Euthanasia decisions were made on the basis of prognosis. The Florida specimens were obtained from deceased dolphins found off the Florida coast, either beached or floating. Necropsies on these dolphins were performed at the Hubbs Seaworld Research Institute. These specimens were obtained with permission from the National Marine Fisheries Services (S1 Table).

At necropsy, external and internal examinations were performed and tissue samples were collected for histopathology and infectious disease testing (Morbillivirus, *Brucella* spp., etc. [48]) where available (S1 Table). Brains from the Massachusetts’ cohort (n = 7 dolphins) were harvested, and a mid-sagittal cut was made to separate each brain into right and left hemispheres. One hemisphere was frozen and stored in an ultra-low freezer at -80°C for toxicological analysis and the other hemisphere was fixed in 10% neutral buffered formalin for one month for histopathological assessments. Cryopreserved brain tissues were sampled for analytical measures of BMAA (see methods below). Formalin-fixed brain hemispheres were used to assess neuropathological changes (see methods below). Toxicological and histological assessments of dolphin brain specimens were performed with the approval from the NOAA Southeast Region Stranding Program and National Marine Fisheries Service.

### Analytical reagents

BMAA, a non-protein amino acid, was measured in dolphin brain tissue using fluorescence high performance liquid chromatography and ultra-performance liquid chromatography/ tandem mass spectrometry (UPLC-MS/MS) as described in Mondo *et al*. 2012 & 2014 [26,49]: AQC Waters AccQTag reagent (PN WAT052880) Reagent Kit was purchased from the Waters Corporation (Milford, MA, USA). Acetonitrile (ACN), hydrochloric acid (HCl) and HPLC grade water, were acquired from VWR (Suwanee, GA, USA). β-N-methylamino-L-alanine hydrochloride (BMAA) DL-2,4-diaminobutyric acid dihydrochloride (DAB)were purchased from Sigma Aldrich (B-107 and 32830, respectively, St. Louis, MO, USA). N-2(amino)ethylglycine (AEG) was obtained from TCI America (A1153, Portland, OR).

### Brain extraction and analytical methods

Frozen punch biospecimens (200 mg) from the cerebral cortex of each dolphin were made using a 6.0mm Biopunch (Ted Pella, Inc., Redding CA, USA). Punch biospecimens were obtained from the auditory cortex (ACtx) and the visual cortex (VCtx). Following sampling, tissue biospecimens were subjected to total acid hydrolysis and assayed for total concentrations of BMAA and BMAA structural isomers N-(2-aminoethyl) glycine (AEG) and 2,4-diaminobutyric acid (DAB) as described by Mondo et al 2012 [26]. No attempts were made to differentiate free from protein bound BMAA. Briefly, a 1:6 W/V of 6 N HCl (1000 μL) was added to each cortical biopsy, followed by sonication. Samples were then hydrolyzed on a heat block for 18 hours at 110°C, filtered at 13,800 x g for 3 minutes at 4°C (0.22 lm Ultrafree-MC, Millipore, Bedford, MA, USA), and concentrated in a speed-vacuum (Thermo-Savant SC250DDA Speed Vac Plus with a Savant refrigerator trap RVT 4104). The dried extract was then resuspended to 1000 μL of 20 mM HCl. An aliquot of the sample extract was derivatized with AQC using the AccQ-Fluor reagent (Waters Crop, Milford, MA). The derivatized samples (20 μL resuspended HCl extract, 60 μL of borate buffer, and 20 μL AccQ-Tag) were run in parallel with buffer and AQC blanks, and L-BMAA, AEG, DAB and reference amino acid standards. Since control dolphin specimens could not be obtained, fresh frozen human brain tissues were used in our assay. For a negative control, frozen punch biopsies were obtained from the cortex of a 68 year-old human female with no known neurological changes and previously found to be free of BMAA [18]. For a positive control, punch biopsies were obtained from a 77 year-old human male with ALS whose tissues were previously shown to contain a high concentration of BMAA [18]. In addition, control sample matrixes were also spiked with known amounts of BMAA to confirm the efficiency of extraction.

### HPLC method

BMAA and its structural isomers (AEG & DAB) were detected and quantified using a validated HPLC method [50,51] with minor modifications [19,26]. Briefly, the non-protein amino acid BMAA was separated from its structural isomers and other protein amino acids using reverse-phase elution (Waters Nova-Pak C18 column, 3.9 mm x 300 mm) on a Waters 1525 Binary HPLC pump and a Waters 717 autosampler. The mobile phase consisted of 140 mM sodium acetate, 5.6 mM triethylamine, pH 5.7 (Eluent A), and 52% (v/v) aqueous acetonitrile (Eluent B) using a flow rate of 1.0 mL/minute and a 10 μl sample injection volume. The samples were eluted using a 60 min gradient: 0.0 minute 100% A; 2.0 minutes 90% A; 5.0 minutes 86% A; 10.0 minutes 86% A; 18.0 minutes 73% A; 30.0 minutes 57% A; 35.0 minutes 40% A; 37.5 minutes 100% B; 47.5 minutes 100% B; 50.0 minutes 100% A; 60.0 minutes 100% A. Samples were derivatized with the AQC fluorescent tag using 20 μL aliquot of sample plus 20 μL 6-aminoquinolyl-N-hydroxysuccinimidyl carbamate (AQC) in 60 μL of borate buffer. Compounds were clearly separated with AEG at 29.6 min, BMAA elution at 31.1 min, and DAB at 33 minutes.

Detection was achieved using a Waters 2475 Multi k-Fluorescence Detector with excitation at 250 nm and emission at 395 nm. Measurements of BMAA in dolphin cortical tissues were compared with those of the human positive and negative controls (described above), and those spiked with known amounts of a reference BMAA standard (Sigma B-107; >95% purity, St. Louis, MO, USA). The limits of detection (LOD) and the limit of quantification (LOQ) were based on the standard deviation (SD) of response and slope (S), calculated from linearity of the response of BMAA. The LOD (2.7 ng) and LOQ (7.0 ng) were obtained by using formula (3.3 x SD)/S and (10 x SD)/S, respectively. The efficiency of the analyte recovery was estimated adding known amounts of a BMAA standard spiked into a reference sample that was below the LOD.

### UPLC-MS/MS method

BMAA and its structural isomers AEG and DAB were separated, detected and quantified by ultra-performance liquid chromatography/ tandem mass spectrometry (UPLC-MS/MS) using a fully validated method as previously described [52,53]. In brief, punch biospecimens of cerebral cortex (200 mg) were weighed and suspended in 1.0 mL of 6 N HCl sealed with N_2_ gas blown into the tubes for 30 s to displace oxygen. Brain samples were hydrolyzed for 18 hours at 110 °C. A subsample of 400 μL was filtered (0.22 μm PVDF Ultrafree MC centrifuge filters; EMD Millipore; Billerica, MA, USA) and a 100 μL aliquot was dried overnight (Labconco Centrivap; Kansas City, MO, USA). The sample was reconstituted in 1.0 mL 20 mM HCl and derivatized as described above. BMAA, AEG and DAB were separated by reverse phase C18 chromatography (BEH column 150 × 2.1 mm 1.7 μm; Waters) and eluted with a gradient of 20 mM ammonium formate with 0.2% formic acid (A) and 0.1% formic acid in acetonitrile; (B). Gradient was delivered by a Waters Acquity I-Class UPLC (Milford, MA, USA) (0 min, 95% A; 1.0 min, 95% A; 7 min, 85% A; 7.5 min, 78% A; 8 min, 15% A; 8.5 min, 15% A; 8.6 min, 95% A; 10 min, 95% A) with a flow rate of 0.7 mL/min at 52 °C. Compounds were clearly separated with BMAA elution at 6.56 min (%RSD = 0.23), AEG at 6.67 minute (%RSD = 0.22) and DAB at 6.82 minutes (%RSD = 0.26). Triplicate measures were performed on each dolphin sample. Ions were detected on a triple quadrupole tandem mass spectrometer (Waters Xevo TQS, Milford, MA, USA) with the following parameters: cone voltage was 16 V. Capillary voltage was set to 2500 V with a source offset of 50 V. Desolvation temperature was 550 °C, with a corresponding gas flow of 800 L/hour and a cone gas flow of 150 L/hour. Collision-induced-dissociation was performed with 99.99% pure argon pressurized to 7.0 bar with a dwell time of 0.05 sec. The characteristic transitions were detected as: BMAA 459 > 258 at collision voltage 18 V, DAB 459 > 188 at collision voltage 20 V, AEG 459 > 214 at collision voltage 20 V. The method performance characteristics were determined by definitions of the Environmental Protection Agency (EPA). The Method Detection Limit (MDL) was < 0.01 pg on column. The lower Limit of Quantification was > 0.02 pg on column. The average % recovery of BMAA in triplicate samples spiked at 80%, 100% and 120% of the expected value was 98.3%. The precision was <6% relative standard deviation (%RSD) for all replicated analyses in the sample matrix. The HorRat value was calculated for triplicate analyses and falls within the AOAC recommended range of 0.5–2.0 [52].

### Immunohistochemistry

To investigate neuropathological changes, formalin-fixed brain tissue blocks (2 x 2 x 0.2 cm^3^) were collected from the ACtx, VCtx and medulla (Md) from seven dolphins. Tissue blocks were processed through xylenes and alcohols and embedded in paraffin wax. Microtome sections were cut at 5 μm and mounted on 1 x 3 inch glass slides. Tissue sections were deparaffinized in three changes of xylene for 10 minutes each, followed by two changes of absolute ethanol for 5 minutes each, then 95% ethanol for 5 minutes. Tissues sections were stained with hematoxylin and eosin (H&E) (for cytoarchitecture) and thioflavine-S (for plaques, tangles, and dystrophic neurites). Modified Bielschowsky’s (MB) silver staining was performed at AML laboratories using an American MasterTech stain kit (Lodi, CA, USA) [54].

To probe for the specific protein antigens noted below, after rehydration sections were incubated in 3% H_2_O_2_ in methanol for 10 minutes, followed by rinsing in 3 changes of distilled water (DiH_2_O) each for 5 minutes. Slides were then incubated in 98% formic acid for 45 seconds, followed by a wash in 3 changes of DiH_2_O on a Thermolyne Roto Mix shaker, and incubation in phosphate-buffered saline pH 7.4 (PBS) for 5 minutes. To block non-specific antibody bindings, 10% normal donkey serum in PBS pH 7.4 (PBS) was applied to tissue sections in a humidity chamber and incubated at room temperature for 30 minutes. Sections were then probed with mouse anti-β-amyloid (1:800, Covance, Princeton, NJ, USA); anti-neuronal nuclei protein (Anti-NeuN Antibody, clone A60; 1:200, Millipore, MA, USA); or anti-glial fibrillary acidic protein (GFAP; 1:200, DAKO/Agilent, CA, USA). Antibodies were allowed to incubate at 4°C overnight, and then the slides were rinsed with PBS for 3 x 10 minutes, followed by additional application of 2% normal donkey serum for 10 minutes then PBS rinse. A donkey anti-mouse-biotin (1:200; Jackson Immunoresearch, West Grove, PA, USA) conjugated goat anti-mouse secondary antibody was incubated on tissue sections for 2 hours at room temperature, rinsed with PBS wash for 10 minutes followed by application of ExtrAvidin peroxidase (1:5,000, Sigma-Aldrich, St. Louis, MO, USA) in PBS for incubation for 1 hour. ExtrAvidin peroxidase was detected using DAB solution (100 mL DAB = 98 mL PBS + 2 mL 25mg/mL DAB + 16.6 μL 3% H_2_O_2_) for 10 minutes. Slides were then washed in two changes of PBS, rinsed with distilled water, counterstained with Gill No. 1 Hematoxylin for 20 seconds, and rinsed under running tap water for 5 minutes. Frontal cortex samples from a patient with advanced AD were used as a positive control for all neuropathology markers listed above.

### Digital and fluorescent microscopic pathology

Histology slides were scanned at 40x resolution using a TissueScope LE (Huron Digital Pathology, Waterloo, ON, CAN). Digital scanning allowed for complete micropathological review of the entire dolphin brain tissue sections and margins at an optimal resolution of (0.2 μm/pixel at 40x). High quality tif file images (975 x 477 pixels) were exported from TissueScope LE and imported into NIH ImageJ 64 VER1.44o (NIH, Bethesda, Maryland, USA) for analysis of Aβ^+^ density and Aβ^+^ plaque area. To determine number and area size of Aβ^+^ plaques, a total of 6 images were analyzed per dolphin (ACtx n = 3 scans and VCtx n = 3 scans). Aβ^+^ plaques were annotated and quantified using ImageJ cell counter plug-in software and presented as the mean and standard error of Aβ^+^ deposits per 5x scan. A threshold was applied to regions of interest to determine the area of each Aβ^+^ plaques. Slides stained with MB silver, thioflavine-S, 4',6-diamidino-2-phenylindole (DAPI), GFAP and Neu-N were visualized at 20x magnification using a Zeiss Apotome fluorescent microscope (USA).

### Statistics

Statistical analyses were performed using Prism Version 7 software (Graph Pad, La Jolla, CA, USA). Single comparisons tests were analyzed using Mann-Whitney U and Wilcoxon matched-pairs test. Multiple comparisons were analyzed using Friedman test with Dunn’s multiple comparison test. For correlation analyses, the Pearson’s Correlation Coefficient and Spearman correlation tests were used to determine significance. All data and results were expressed as the Mean ± Standard Error; significance level of alpha = 0.05. Due to the limited size of our sample cohort, Cohen’s d was used to determine effect size and D'Agostino & Pearson test was used to determine normality of data. A table of descriptive statistics for each comparison can be found in supplementary material (S3 Table).

## Results

### Stranded dolphins

A total of fourteen dolphins were investigated from the following dolphin species: bottlenose dolphin (*Turiops truncates*) and common dolphin (*Delphinus delphis*) (S1 Table). Nine dolphins (64%) in our sample cohort were observed dead. Five dolphins (36%) were part of a mass stranding. Four dolphins (29%) had conditions that have been shown to cause strandings: one with mechanical trauma (Hubbs 0805 Tt) and three cases of *Brucella* infections (IFAW 12-223Dd, 12-228Dd and 12-229Dd) [55]. Seven dolphins (50%) were female; seven dolphins were male (50%). One dolphin (7%) was pregnant at the time of death. Nine dolphins (64%) in the study were categorized as adult and five as sub-adult (36%). The bottlenose dolphins had 1.3-fold greater mean length than the common dolphins (*P*<0.0001, *t*-Test) (S1 Table). Bottlenose dolphins were also 4-fold greater in weight than the common dolphin (*P*<0.0001, *t*-Test) (S1 Table). All dolphins except for two (Hubbs 0805-Tt and Hubbs 0630-Tt) had no detectable injury resulting from human interaction. Dolphin Hubbs 0805-Tt had an old scar on her dorsal fin from a boat injury. Dolphin Hubbs 0630-Tt had fishing gear entangled around the flukes and hooks in the oral cavity. External examinations of several dolphins (29%) showed signs of stranding stress (IFAW12-201Dd), cauliflower-like lesions on flukes, peduncle and dorsal fin (IFAW12-228Dd), a large circular wound (Hubbs 0805 Tt) and several broken ribs (Hubbs 0541-Tt). One dolphin (7%) (IFAW12-198Dd) had incidental post-stranding injuries to the eyes as a result of gull pecking. Gross examinations of internal structures were unremarkable, except for two dolphins (14%) with non-specific lung (Hubbs 0541 Tt) and spleen (IFAW12-198Dd) pathology. One dolphin had signs of hepatitis (IFAW12-201Dd) [48].

### Detection of BMAA

**Fig 1:**
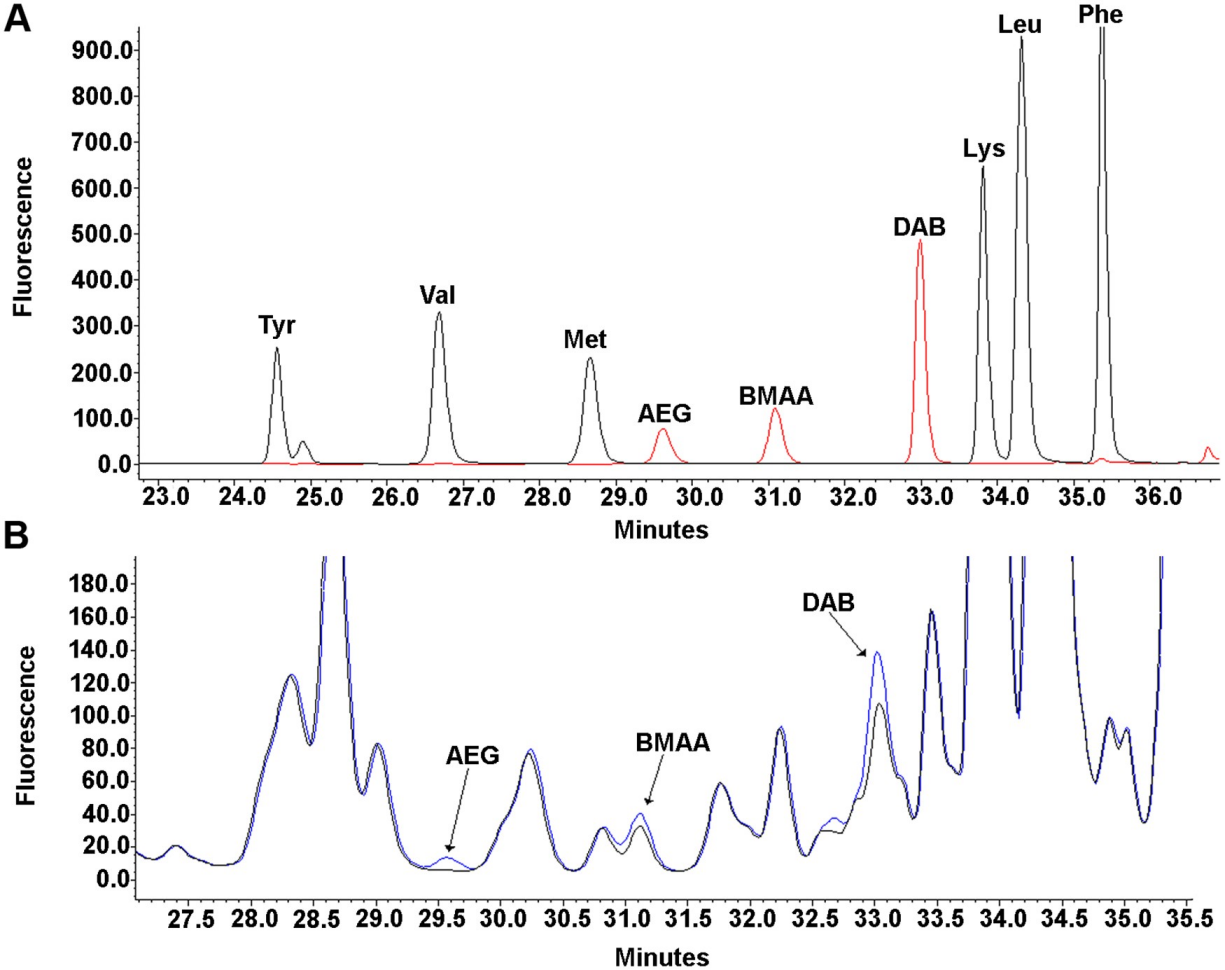
HPLC-FD detection of BMAA in the cerebral cortex of stranded dolphins. (A) Separation of 6-aminoquinolyl-N-hydroxysuccinimidyl carbamate (AQC) derivatized amino acid standards tyrosine (Try), valine (Val), methionine (Met), Lysine (Lys), Leucine (Leu) and Phenylalanine (Phe). BMAA and BMAA structural isomers N-(2-aminoethyl)-glycine (AEG) & 2,4-diaminobutyric acid (DAB) standards are shown in red. (B) Representative chromatogram from the visual cortex (VCtx) of stranded dolphin IFAW12-201Dd with low-concentration spikes of BMAA and BMAA isomer standards in the same dolphin sample shown in blue. Chromatogram shows BMAA has a distinct peak with a retention time of 31.1 minutes. AEG and DAB have distinct retention times of 29.6 and 33.0 minutes, respectively.

BMAA was detected in 13 of the 14 dolphins (93%) beached in both Florida and Massachusetts (Table 1, S1 Table, Fig 1). One dolphin injured by boat collision was negative for BMAA (Hubbs 0805 Tt). BMAA levels in the other 13 dolphin brains ranged from (20–748 μg/g). The overall mean concentration of BMAA was 287.9 ± 64.3 μg/g and the median concentration was 170 μg/g. The bottlenose dolphins that stranded in Florida had 3-fold (*P* = 0.01; *t*-Test) higher concentrations of BMAA than the common dolphins from Massachusetts (Table 1, S1 Table). Sex had no effect on the mean concentration of BMAA. The average BMAA/length of dolphin was 1.2 ± 0.2 μg/g/cm. BMAA concentrations were positively correlated with the length of dolphin (n = 13; Pearson r = 0.6002; *P* = 0.01) (S3 Table). The mean concentration of BMAA in the stranded dolphin brains was 1.4-fold higher than in reference brains of patients with AD and ALS (S1 Table) [18]. In some dolphins, BMAA levels detected were up to 3.6-fold greater than found in those of AD and ALS reference brains (S1 Table). The levels of BMAA and the BMAA isomers (AEG and DAB) were not region-specific and the mean concentration of BMAA, AEG, and DAB did not differ in auditory or visual cortex (Table 2). DAB was detected in 7 of 7 (100%) of dolphins from Massachusetts. The mean concentration of DAB detected is 2.4-fold higher than BMAA averaged across all dolphins. AEG was detected in 6 of 7 (86%) of dolphins stranded in Massachusetts. The mean concentration of AEG was 4-fold lower than BMAA and 9.4-fold lower than DAB (*P*<0.0001; ANOVA). The detection of BMAA and BMAA structural isomers in the dolphin brain was confirmed using LC-MS/MS in representative animals sampled from the cohort (S4 Table).

**Table 1:** Comparison of BMAA detected in brains of stranded dolphins.

Agency ID	Necropsy Findings	Sex	Age Class	BMAA (μg/g)
**Bottlenose Dolphin (FL)**				
Hubbs 0805 Tt	Boat Injury	Female	Adult	*ND*
Hubbs 0720 Tt	Unknown	Female	Adult	114
Hubbs 0717 Tt	Unknown	Male	Adult	295
Hubbs 0630 Tt	Unknown	Female	Adult	335
Hubbs 0541 Tt	Unknown	Male	Adult	541
Hubbs 0636 Tt	Unknown	Male	Sub-Adult	675
PCNMF S08-01 Tt	Unknown	Female	Adult	748
Mean ± SE				451 ± 99 **
Min—Max				114–748
**Common Dolphin (MA)**				
IFAW 12–228 Dd	Brucellosis	Male	Adult	20
IFAW 12–223 Dd	Brucellosis	Male	Sub-Adult	111
IFAW 12–200 Dd	Unknown	Male	Sub-Adult	127
IFAW 12–198 Dd	Unknown	Female	Adult	129
IFAW 12–229 Dd	Brucellosis	Male	Sub-Adult	157
IFAW 12–205 Dd	Unknown	Female	Sub-Adult	170
IFAW 12–201 Dd	Unknown	Female	Adult	320
Mean ± SE				147 ± 34
Min—Max				14–320

**FL**, Florida; **MA**, Massachusetts;

**, *P =* 0.01 (*t*-Test);***ND***, Not Detected; ***± SE***: Standard Error

**Table 2:** Region specific detection of BMAA and BMAA Isomers in the brains of stranded dolphins.

Agency ID	ACtx			VCtx		
	BMAA (μg/g)	DAB (μg/g)	AEG (μg/g)	BMAA (μg/g)	DAB (μg/g)	AEG (μg/g)
IFAW 12–228 Dd	20.2 ± 0.3	106.4 ± 1.1	NQ	ND	97.7 ± 0.0	ND
IFAW 12–223 Dd	52.6 ± 0.6	210.8 ± 0.6	NQ	168.5 ± 0.1	411.1 ± 1.0	50.2 ± 0.7
IFAW 12–229 Dd	120.6 ± 1.0	300.0 ± 4.0	NQ	193.3 ± 1.0	419.5 ±1.1	31.6 ± 0.3
IFAW 12–205 Dd	136.20 ± 1.5	333.7 ± 5.8	NQ	204.4 ± 0.6	461.8 ± 4.3	42.9 ± 3.1
IFAW 12–198 Dd	193.7 ± 5.1	439.9 ± 9.5	NQ	63.9 ± 0.2	244.2 ± 0.4	27.6 ± 1.4
IFAW 12–200 Dd	209.6 ± 2.1	465.0 ± 7.9	42.2 ± 1.0	43.7 ± 0.5	212.9 ± 2.6	17.2 ± 1.7
IFAW 12–201 Dd	328.4 ± 3.5	741.8 ± 7.0	73.5 ± 1.7	312.2 ± 0.9	644.6 ± 3.1	ND
**Mean ± SE**	151.6 ± 39.2	371.1 ± 77.6***	57.9 ± 15.7	164.3 ± 40.4	356.0 ± 69.2***	33.9 ± 5.8
**Min—Max**	20–328	106–742	42–74	44–312	98–645	17–50

***ND***, Not Detected; ***NQ***, Not Quantifiable; ***± SE***: Standard Error;

***, *P*<0.0001 (ANOVA)

### Neuropathology in stranded dolphins

**Fig 2:**
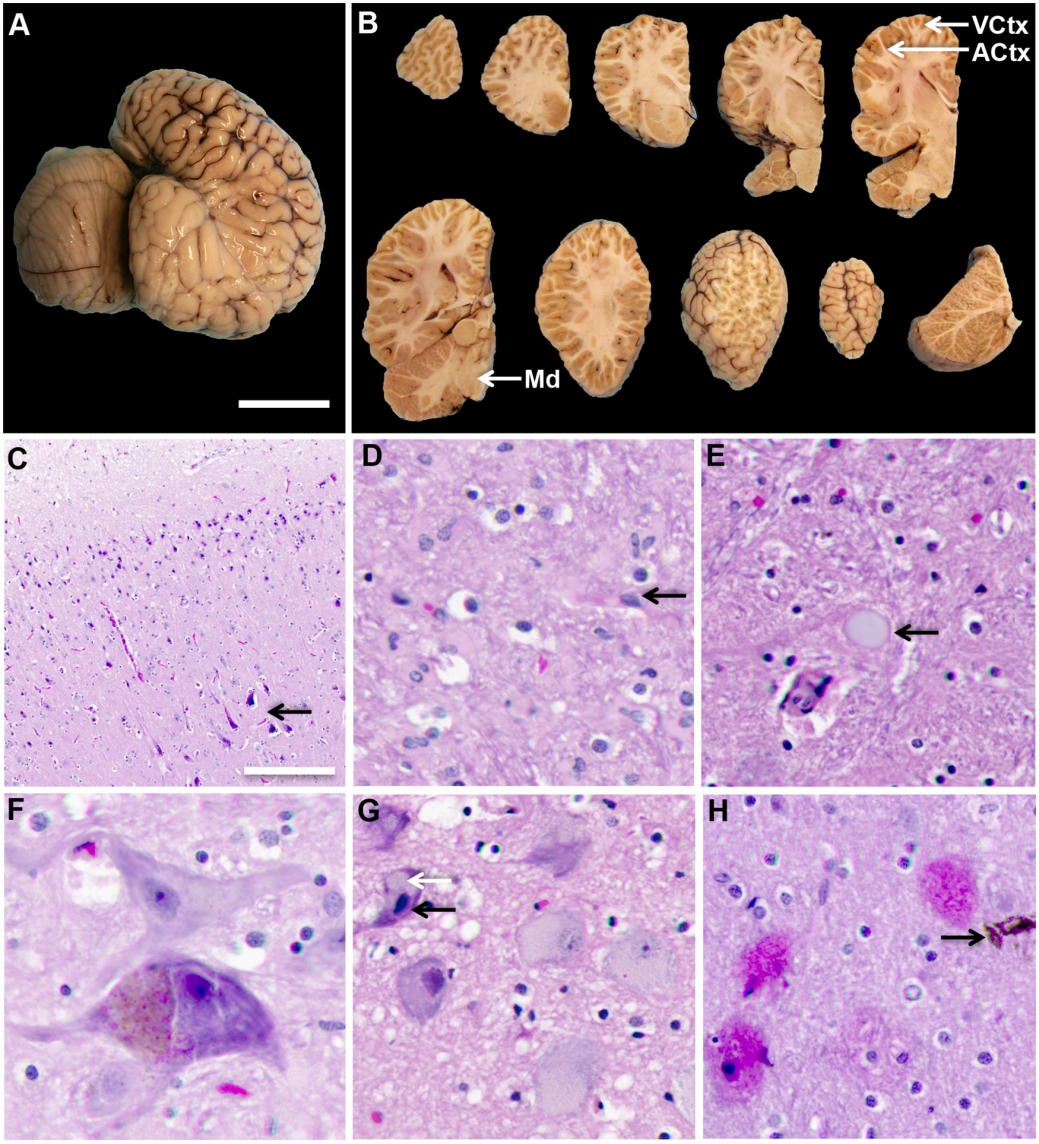
Gross and microscopic evaluation of postmortem brains from stranded dolphins. (A) External examination was performed on the cerebral cortex and cerebellum of formalin-fixed hemispheres from stranded dolphins (n = 7). (B) Following external examinations, brain hemispheres were cut into a series of coronal slices to investigate internal gray and white matter structures. Tissue blocks were sampled from anatomical regions in the dolphin cerebral cortex and brainstem involved with acoustico-motor navigation: auditory cortex (ACtx), visual cortex (VCtx), and the medulla oblongata (Md). (C) Digital pathology scans were obtained from routine histological stain. H&E stain shows hypoxic and eosinophilic changes in neurons of both upper and lower cortical layers. (D) Gliosis was also observed in the cerebral cortex. (E) Advanced age-related changes were observed including, corpora amylacea and (F) lipofuscin granules. (G) Karyorrhexis nuclear changes (black arrow) and chromatolysis (white arrow) were observed. (H) Representative scans of eosinophilic plaques and a rare hemosiderin deposits were observed in the ACtx of stranded dolphins. Representative scale bar: 5 cm (A, B), 1000 μm (C), 200 μm (D, F, G), 50 μm (E, H).

Gross examinations of external brain structures were unremarkable for all stranded dolphins examined. Brain structures showed normal anatomic landmarks with normal gyral and sulcal patterns (Fig 2A). No atrophy, white matter lesions, infarcts or hemorrhages were observed on gross inspection of coronal sections of the brain. Internal gray and white matter structures were also unremarkable, with clear delineation. The lateral, third and fourth ventricles were normal in size (Fig 2B). Microscopic examination of H&E sections from the ACtx, VCtx and Md tissue sections revealed evidence of cellular injury and age-related changes (see below). Microscopic examination showed hypoxic, shrunken, and eosinophilic neurons in upper and lower cortical layers and within brainstem nuclei (Fig 2C). Gliosis, mild neuronal satellitosis and neuronal atrophy were present in cortical layers and brainstem (Fig 2D). Age-related histological changes included accumulation of lipofuscin, chromatolysis, and corpora amylacea (Fig 2E–2G). In addition, moderate numbers of eosinophilic plaques were seen (Fig 2H) and edema was observed throughout parts of the cerebral cortex and brain stem of all dolphins (Fig 2C–2H). The pathological changes described above were present in all dolphins in varying degrees regardless of age, sex or BMAA concentrations.

### Aβ^+^ Pathology

**Fig 3:**
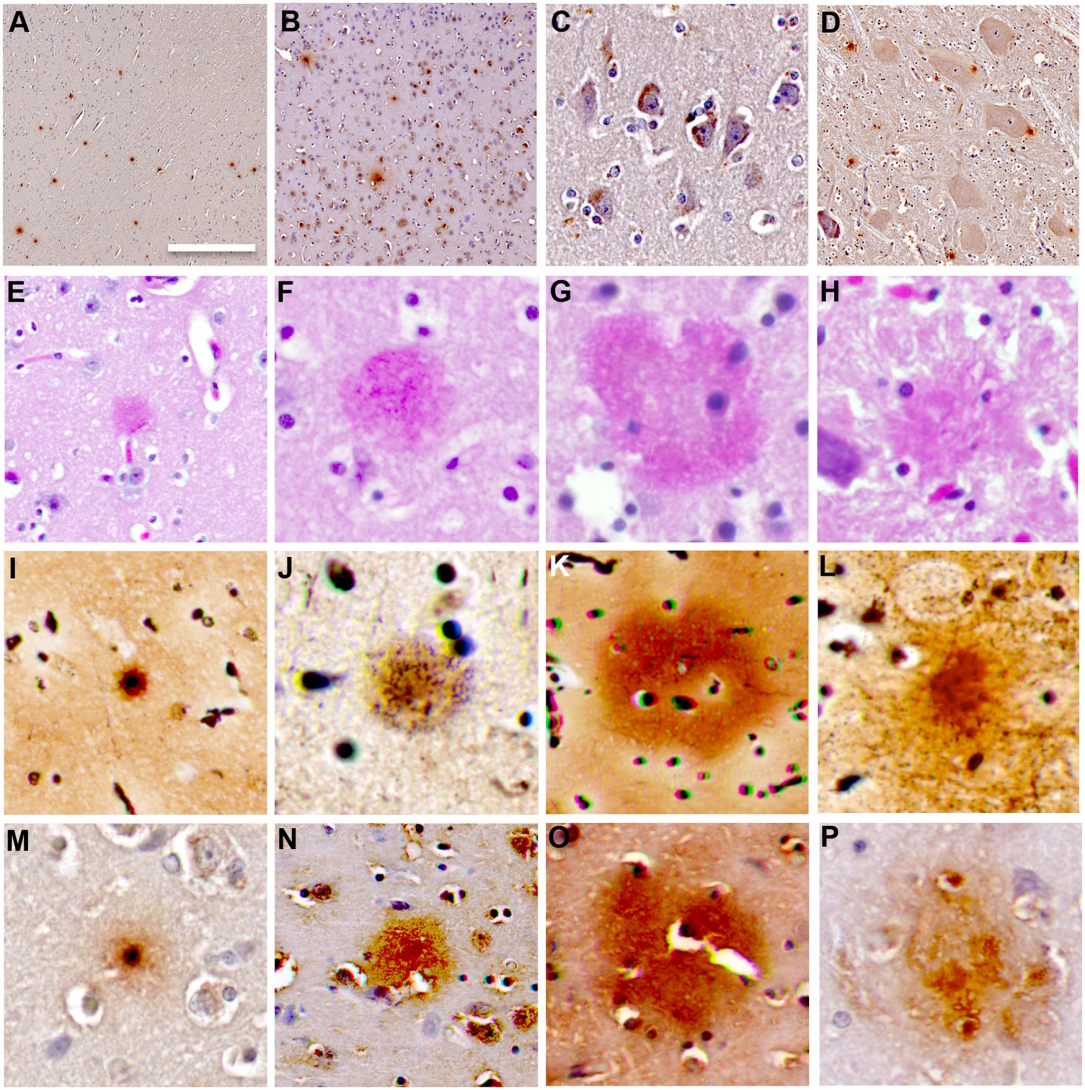
Aβ deposition in the cerbral cortex of stranded dolphins. (A) Anti-Aβ IHC demonstrates Aβ^+^ plaques in the cerebral cortex of stranded dolphins. (B) Intraneuronal Aβ^+^ accumulation was aslo observed throughout upper and lower cortical layers. (C) High-resolution digital patholgy scans of pyramidal neurons in the ACtx containing dense intracellalur Aβ^+^ inclusions. (D) Large and sparse Aβ^+^ plaques were observed in the Md of stranded dolphins. (E-P) Aβ^+^ plaques were different in morphology as observed by H&E staining (E-H), MB silver staining (I-L) and Aβ^+^ IHC (M-P). Aβ^+^ plaques ranged from small focal with compact cores (E, I, M), primative immature cotton wool-like (F, J, N), to large and diffuse (G, K, O) and ill-defined (H, L, P). Representative scale bar: 500 μm (A, B & D), 100 μm (C), 50 μm (E-P).

**Fig 4:**
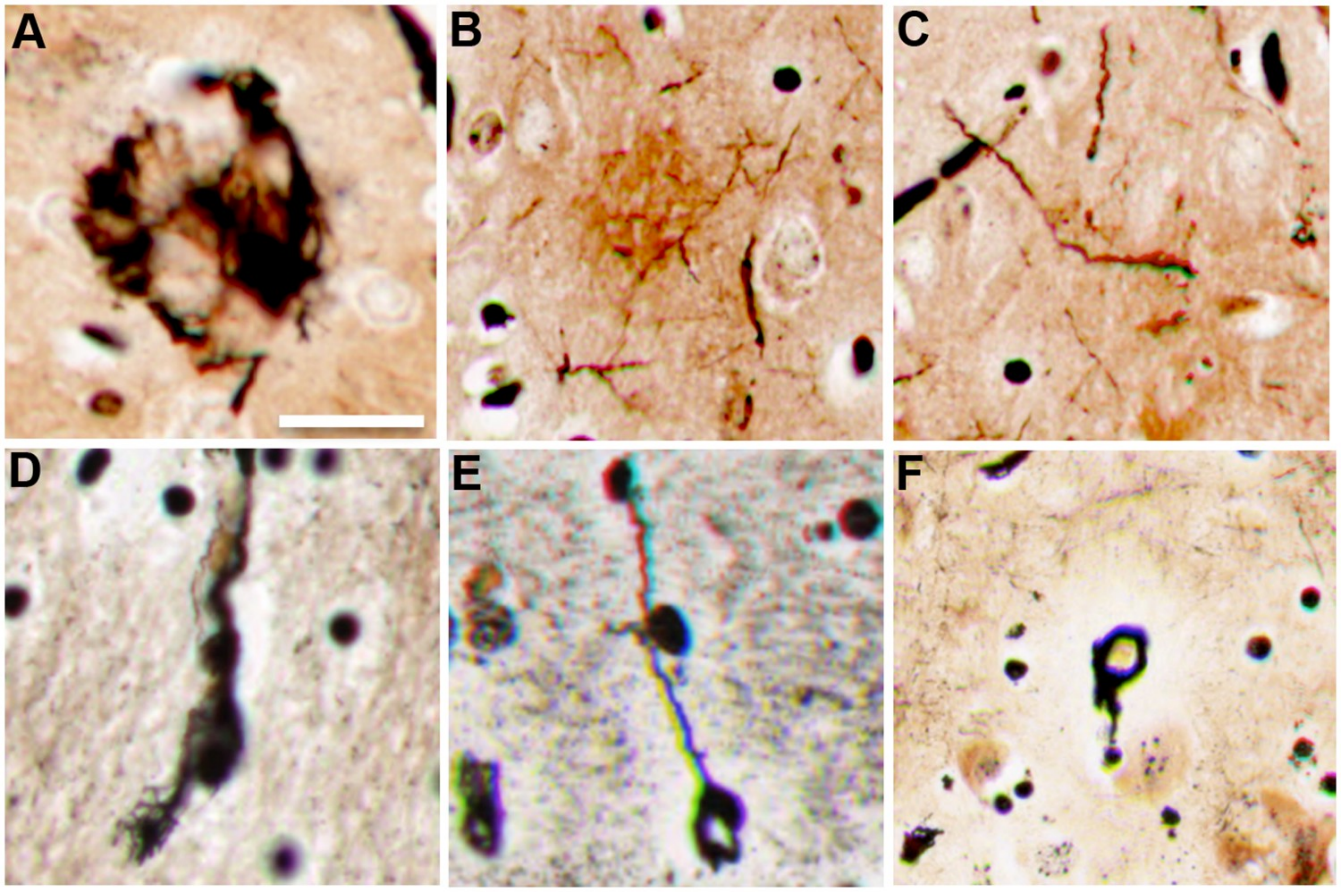
Neurodegenerative changes observed in the brains of stranded dolphins. (A) MB silver staining illustrates AD-like compact neuritic plaque in the ACtx of a stranded dolphin. (B) A representative digital scan of an ill-defined agrophyllic plaque containing dystrophic neurites. (C) Dense neuropil threads were observed in the VCtx. (D, E) agrophyllic neurons containing dystrophic neurites. (F) Coiled body in the ACtx. Representative scale bars: 50 μm.

**Fig 5:**
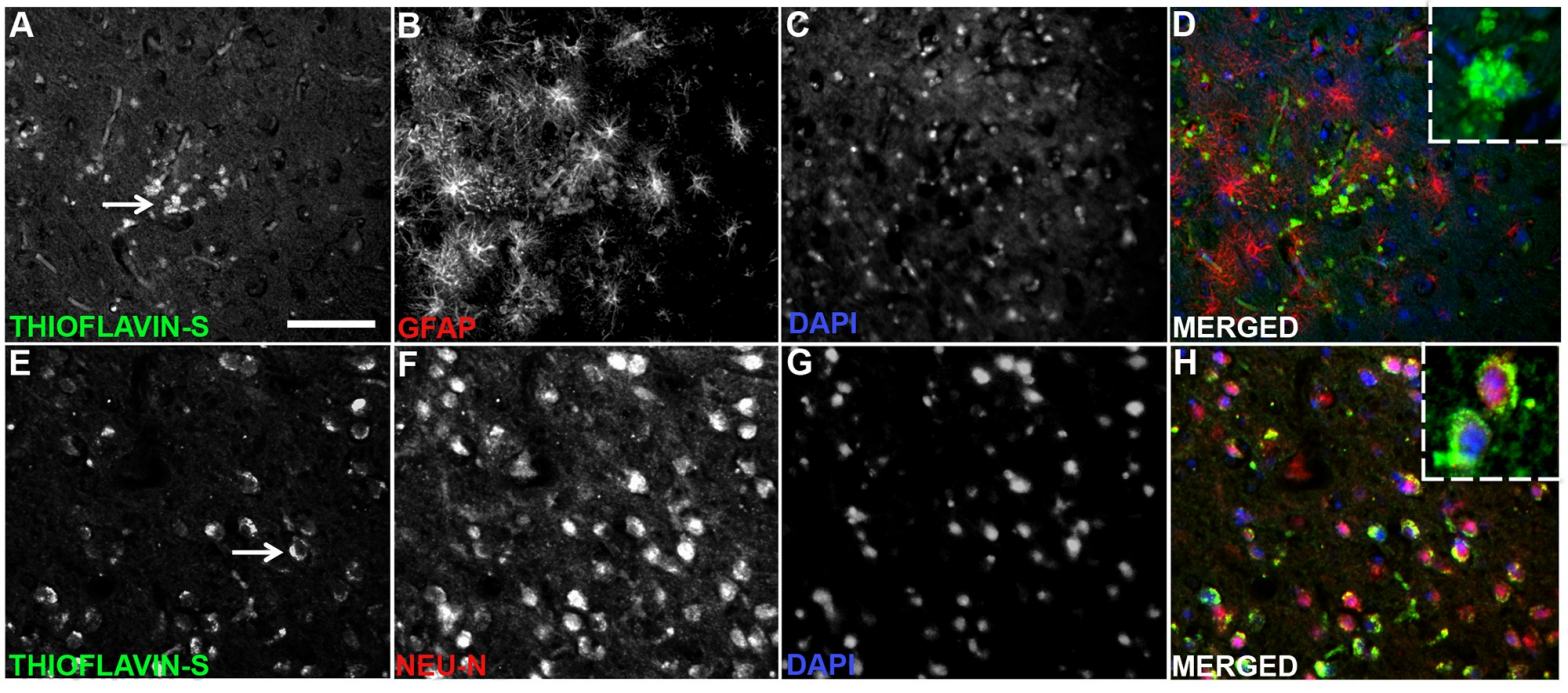
Thioflavin-S^+^ pathology in the auditory cortex of stranded dolphins. Thioflavin-S^+^ staining and IHC with GFAP and Neu-N was used to determine co-localization of plaques and intracellular inclusions with astroglia and neurons in the stranded dolphin brain. (A, E) Thioflavin-S^+^ plaques and intracellular staining was observed in the ACtx (arrows). (B, F) Activated GFAP^+^ astrocytes are demonstrated surrounding a cluster of dense thioflavin-S^+^ plaques. Neu-N^+^ neurons are shown with intracellular thioflavin-S^+^ inclusions. (C, G) DAPI staining highlights intact cellular nuclei. (D, H) The merged panels show the relative co-localization of thioflavin-S^+^ plaques, blood vessels, and intracellular inclusions within astrocytes and neurons. Panel inserts (dotted lined boxes) show high magnification detailing thioflavin-S^+^ structures. Representative scale bar: 150 μm.

Anti-Aβ staining showed Aβ^+^ plaques, ranging in occurrence from rare to very frequent, and intracellular localization of Aβ in the cerebral cortex of all stranded dolphins (Fig 3A–3C). Aβ^+^ plaques were observed throughout the ACtx and VCtx. Rare to sparse Aβ^+^ plaques were localized adjacent to neurons in the Md region of the brainstem (Fig 3D). The Aβ^+^ plaques varied from focal clusters of well-defined plaques to diffused and poorly organized plaque-like structures. Aβ^+^ plaques were observed on both routine H&E and MB silver stained sections (Fig 3E–3P). Most Aβ^+^ plaques were senile (diffused) plaques, but rare argophyllic compact neuritic plaques and neuropil threads were observed in the cerebral cortex (Fig 4A–4C). Intracellular silver staining neurofibrillary tangles were observed in cortical neurons with dystrophic neurites, indicating the presence of intracellular inclusions of insoluble proteins (Fig 4D–4F). Thioflavin-S staining was used to confirm the Aβ^+^ staining of plaques and neuronal inclusions (Fig 5A–5H). GFAP^+^ fluorescent immunohistochemistry (IHC) provided evidence of reactive astrocytes around thioflavin-S^+^ plaques (Fig 5A–5D). Semi-quantitative analysis showed the densities of Aβ^+^ plaques were positively correlated in the ACtx and VCtx (n = 7; r = 0.89; *P*<0.05) but, was 1.3-fold denser in the ACtx than in the VCtx (*P* = 0.01; *t*-Test) (Table 3, S3 Table). Dolphins with *Brucella* spp. infections had fewer Aβ^+^ plaques when compared to dolphins without *Brucella* spp. infections (*P* = 0.02; *t-*Test) (S3 Table). The size of the Aβ^+^ plaques did not differ between the two cortical regions. Neither age group nor sex of dolphins affected the Aβ^+^ plaque deposition (S3 Table). Nor was Aβ^+^ plaque deposition correlated with brain BMAA concentration (S3 and S5 Tables).

**Table 3:** Region specific quantification of Aβ^+^ plaques in the stranded dolphin brain.

Agency ID	ACtx Plaques/ Field	VCtx Plaques/ Field	ACtx Plaque Area (Px)	VCtx Plaque Area (Px)
IFAW12-228 Dd	41.3 ± 7.7	25.0 ± 2.2	84.0 ± 9.8	79.6 ± 8.2
IFAW12-229 Dd	42.7 ± 7.9	37.0 ± 6.7	78.7 ± 14.8	90.3 ± 6.8
IFAW12-201 Dd	54.3 ± 2.8	46.0 ± 6.1	55.4 ± 6.6	67.82 ± 9.5
IFAW12-223 Dd	54.7 ± 9.8	30.0 ± 2.4	125.9 ± 34.8	107.16 ± 15.0
IFAW12-198 Dd	77.7 ± 32.3	66.7 ± 9.7	82.0 ± 8.1	102.16 ± 14.7
IFAW12-205 Dd	84.3 ± 7.5	84.3 ± 33.4	89.7 ± 9.8	89.0 ± 8.3
IFAW12-200 Dd	101.0 ± 28.5	70.3 ± 13.0	78.6 ± 6.9	81.0 ± 9.6
**Mean** ± **SE** **Min—Max**	65.1 ± 8.6** 41–101	51.3 ± 8.5 6–84	84.9 ± 8.0 55–126	88.1 ± 5.1 68–181

**, *P*<0.01 (Paired *t*-Test); **Px**, Pixels; ***± SE***: Standard Error

## Discussion

As the world’s climate warms, HABs are becoming more frequent, including in eastern China, which has seen some of the largest cyanobacterial blooms on Earth, and North America. Cyanobacteria produce powerful cyanotoxins that impact aquatic and terrestrial life. Exposures to cyanotoxins are a public health concern as they are linked to organ system damage and disease. Examining the levels of the cyanobacterial toxin BMAA in apex predators, such as dolphins and sharks, provides a powerful bio-indicator of the potential for human exposures [26,27,49].

BMAA is produced by cyanobacteria that are sometimes seen as surface blooms, but can occur in the water column and in benthic mats in lakes, shallow estuaries and bays. In marine systems, BMAA enters the food chain *via* crustaceans and bottom-feeding fish, and then accumulates into long-lived apex predators like sharks [26,27]. We now report that another apex predator, dolphins, when exposed to cyanobacteria or diets of crustaceans and fish containing BMAA, bioaccumulate the toxin in their neuroproteins. In our study, BMAA was present in the brains of stranded dolphins at 1.4-fold greater amounts than in the brains of patients with AD and ALS [18]. Dolphins beached in Florida were observed to have nearly 3-fold higher concentrations of BMAA in their brains than dolphins beached in Massachusetts. The differences in BMAA concentration may be due to differences in diet, occurrences of algal blooms, varying phytoplankton species or modes of stranding [56].

The recognition that the very high incidence of neurodegenerative disease Guam ALS/PDC was likely due to the production of BMAA by endophytic cyanobacteria resident in specialized coralloid roots of cycad trees led to the hypothesis that BMAA is a cause of ALS/PDC and non-endemic ALS, and provided a link to neurodegenerative disorders [19,20]. In Guam, cycad seeds contain BMAA and are used to make flour by the indigenous Chamorro. BMAA is also biomagnified up the food chain to animals like pigs, deer and flying foxes that are part of the Chamorro diet [20,50,57]. Once ingested, BMAA can cross the blood-brain barrier and become incorporated into proteins where it is associated with neuropathological changes as seen in Guam ALS/PDC [12,20]. In this paper, we have now shown that BMAA is detectable in brains of dolphins that also show neuropathological changes characteristic of human neurodegenerative disease. Here, BMAA is also shown to accumulate with increasing length and age class. Thus, our data suggest dolphins may provide a naturalistic model of BMAA toxin exposures in marine environments. The effects of this exposure however are not yet clear.

Dolphins have a highly evolved cerebral cortex that underlies a sophisticated sonar navigation system [58]. Previous studies have shown that dolphins have age-related Aβ^+^ deposits in their brains [58,59]. Aβ^+^ plaques, a diagnostic hallmark of AD, were observed to be widespread in the auditory and visual cortex and to a lesser extent the brainstem of the stranded dolphins examined in our study. Numerous Aβ^+^ intracellular inclusions and dystrophic neurites were also observed. The increased deposition of Aβ^+^ plaques with accompanying histological changes (e.g. lipofuscin, corpora amylacea) suggest cellular brain aging. Accumulation of BMAA in the brains of dolphins may exacerbate these age related changes. This observation also raises concern about the potential risk to human health of chronic exposure to BMAA associated with increasingly frequent algal blooms in surrounding lakes and coastal waters.

Lastly, Marine mammals and fish have long been known to concentrate methylmercury (MeHg). Many water bodies have both high concentrations of mercury and frequent algal blooms. High consumption of fish has been linked both to increased blood levels of mercury and to higher incidence of neurodegenerative disease [60]. The co-occurrence of MeHg and BMAA has synergistic neurotoxicity compared to single exposures [61] and has been suggested as a link to dementia [62]. We detected BMAA in all but one dolphin beached in Florida and Massachusetts. Further studies are needed to investigate the synergistic effect of MeHg on the dolphin brain. The potential combined neurotoxicity of BMAA and MeHg underscores the impending impact of climate change on the marine food web.

## Conclusion

We have detected BMAA in the cerebral cortex of stranded dolphins. We also report Alzheimer-like neurodegenerative changes in the brains of dolphins containing BMAA. The presence of BMAA suggests that dolphins provide an excellent sentinel species for toxin exposures in the marine environment.

## Supporting information

S1 Table Stranded dolphin demographics. (DOCX)

S2 Table Surveillance data from the Phytoplankton Monitoring Network obtained in regions of dolphin stranding. (DOCX)

S3 Table Statistical analyses. (DOCX)

S4 Table LC-MS/MS detection of BMAA. (DOCX)

S5 Table Comparison of BMAA and Aβ^+^ plaques in dolphin brain. (DOCX)

## References

[1] Yan X, Xu X, Wang M, Wang G, Wu S, et al (2017) Climate warming and cyanobacteria blooms: Looks at their relationships from a new perspective. Water Res 125: 449–457. 10.1016/j.watres.2017.09.008 28898702

[2] Erdner DL, Dyble J, Parsons ML, Stevens RC, Hubbard KA, et al (2008) Centers for Oceans and Human Health: a unified approach to the challenge of harmful algal blooms. Environ Health 7 Suppl 2: S2. 10.1186/1476-069X-7-S2-S2 PMC2586715 19025673

[3] Glover WB, Mash DC, Murch SJ (2014) The natural non-protein amino acid N-beta-methylamino-L-alanine (BMAA) is incorporated into protein during synthesis. Amino Acids 46: 2553–2559. 10.1007/s00726-014-1812-1 25096519

[4] Lapointe BE, Tomasko DA, and Matzie WR (1994) Eutrophication and trophic state classification of seagrass communities in the Florida Keys. Bulletin of Marine Science 54: 696–717.

[5] Morabito S, Silvestro S, Faggio C (2017) How the marine biotoxins affect human health. Nat Prod Res: 1–11. 10.1080/14786419.2017.1329734 28532167

[6] van Apeldoorn ME, van Egmond HP, Speijers GJ, Bakker GJ (2007) Toxins of cyanobacteria. Mol Nutr Food Res 51: 7–60. 10.1002/mnfr.200600185 17195276

[7] Buratti FM, Manganelli M, Vichi S, Stefanelli M, Scardala S, et al (2017) Cyanotoxins: producing organisms, occurrence, toxicity, mechanism of action and human health toxicological risk evaluation. Arch Toxicol 91: 1049–1130. 10.1007/s00204-016-1913-6 28110405

[8] Nunn PB (2017) 50 years of research on alpha-amino-beta-methylaminopropionic acid (beta-methylaminoalanine). Phytochemistry 144: 271–281. 10.1016/j.phytochem.2017.10.002 29102875

[9] Spencer PS, Nunn PB, Hugon J, Ludolph AC, Ross SM, et al (1987) Guam amyotrophic lateral sclerosis-parkinsonism-dementia linked to a plant excitant neurotoxin. Science 237: 517–522. 3603037 10.1126/science.3603037

[10] Cox PA, Sacks OW (2002) Cycad neurotoxins, consumption of flying foxes, and ALS-PDC disease in Guam. Neurology 58: 956–959. 11914415 10.1212/wnl.58.6.956

[11] Bradley WG, Mash DC (2009) Beyond Guam: The cyanobacteria/BMAA hypothesis of the cause of ALS and other neurodegenerative diseases. Amyotrophic Lateral Sclerosis 10 Suppl 2: 7–20. 10.3109/17482960903286009 19929726

[12] Xie X, Basile M, Mash DC (2013) Cerebral uptake and protein incorporation of cyanobacterial toxin beta-N-methylamino-L-alanine. Neuroreport 24: 779–784. 10.1097/WNR.0b013e328363fd89 23979257

[13] Dunlop RA, Cox PA, Banack SA, Rodgers KJ (2013) The non-protein amino acid BMAA is misincorporated into human proteins in place of l-serine causing protein misfolding and aggregation. PLoS One 8: e75376 10.1371/journal.pone.0075376 24086518 PMC3783393

[14] Main BJ, Italiano CJ, Rodgers KJ (2017) Investigation of the interaction of beta-methylamino-L-alanine with eukaryotic and prokaryotic proteins. Amino Acids. 10.1007/s00726-017-2525-z 29235019

[15] Cox PA, Davis DA, Mash DC, Metcalf JS, Banack SA (2016) Dietary exposure to an environmental toxin triggers neurofibrillary tangles and amyloid deposits in the brain. Proc Biol Sci 283. 10.1098/rspb.2015.2397 PMC4795023 26791617

[16] Cox PA, Davis DA, Mash DC, Metcalf JS, Banack SA (2016) Do vervets and macaques respond differently to L-BMAA? Neurotoxicology In press10.1016/j.neuro.2016.04.017 27133441

[17] Lobner D, Piana PM, Salous AK, Peoples RW (2007) Beta-N-methylamino-L-alanine enhances neurotoxicity through multiple mechanisms. Neurobiol Dis 25: 360–366. 10.1016/j.nbd.2006.10.002 17098435 PMC3959771

[18] Pablo J, Banack SA, Cox PA, Johnson TE, Papapetropoulos S, et al (2009) Cyanobacterial neurotoxin BMAA in ALS and Alzheimer’s disease. Acta Neurologica Scandinavica 120: 216–225. 10.1111/j.1600-0404.2008.01150.x 19254284

[19] Murch SJ, Cox PA, Banack SA, Steele JC, Sacks OW (2004) Occurrence of beta-methylamino-l-alanine (BMAA) in ALS/PDC patients from Guam. Acta Neurol Scand 110: 267–269. 10.1111/j.1600-0404.2004.00320.x 15355492

[20] Murch SJ, Cox PA, Banack SA (2004) A mechanism for slow release of biomagnified cyanobacterial neurotoxins and neurodegenerative disease in Guam. Proc Natl Acad Sci U S A 101: 12228–12231. 10.1073/pnas.0404926101 15295100 PMC514403

[21] Torbick N, Hession S, Stommel E, Caller T (2014) Mapping amyotrophic lateral sclerosis lake risk factors across northern New England. Int J Health Geogr 13: 1 10.1186/1476-072X-13-1 24383521 PMC3922844

[22] Masseret E, Banack S, Boumediene F, Abadie E, Brient L, et al (2013) Dietary BMAA exposure in an amyotrophic lateral sclerosis cluster from southern France. PLoS One 8: e83406 10.1371/journal.pone.0083406 24349504 PMC3862759

[23] Caller TA, Doolin JW, Haney JF, Murby AJ, West KG, et al (2009) A cluster of amyotrophic lateral sclerosis in New Hampshire: a possible role for toxic cyanobacteria blooms. Amyotroph Lateral Scler 10 Suppl 2: 101–108. 19929741 10.3109/17482960903278485

[24] Caller TA, Field NC, Chipman JW, Shi X, Harris BT, et al (2012) Spatial clustering of amyotrophic lateral sclerosis and the potential role of BMAA. Amyotroph Lateral Scler 13: 25–32. 10.3109/17482968.2011.621436 22214351

[25] Torbick N, Ziniti B, Stommel E, Linder E, Andrew A, et al (2018) Assessing Cyanobacterial Harmful Algal Blooms as Risk Factors for Amyotrophic Lateral Sclerosis. Neurotox Res 33: 199–212. 10.1007/s12640-017-9740-y 28470570 PMC5727154

[26] Mondo K, Hammerschlag N, Basile M, Pablo J, Banack SA, et al (2012) Cyanobacterial neurotoxin beta-N-methylamino-L-alanine (BMAA) in shark fins. Mar Drugs 10: 509–520. 10.3390/md10020509 22412816 PMC3297012

[27] Brand LE, Pablo J, Compton A, Hammerschlag N, Mash DC (2010) Cyanobacterial blooms and the occurrence of the neurotoxin beta-N-methylamino-L-alanine (BMAA) in South Florida aquatic food webs. Harmful Algae 9: 620–635. 10.1016/j.hal.2010.05.002 21057660 PMC2968748

[28] Jonasson S, Eriksson J, Berntzon L, Spacil Z, Ilag LL, et al (2010) Transfer of a cyanobacterial neurotoxin within a temperate aquatic ecosystem suggests pathways for human exposure. Proc Natl Acad Sci U S A 107: 9252–9257. 10.1073/pnas.0914417107 20439734 PMC2889067

[29] Brown A, Foss A, Miller MA, Gibson Q (2018) Detection of cyanotoxins (microcystins/nodularins) in livers from estuarine and coastal bottlenose dolphins (Tursiops truncatus) from Northeast Florida. Harmful Algae 76: 22–34. 10.1016/j.hal.2018.04.011 29887202

[30] Twiner MJ, Fire S, Schwacke L, Davidson L, Wang Z, et al (2011) Concurrent exposure of bottlenose dolphins (Tursiops truncatus) to multiple algal toxins in Sarasota Bay, Florida, USA. PLoS One 6: e17394 10.1371/journal.pone.0017394 21423740 PMC3053359

[31] Schwacke LH, Twiner MJ, De Guise S, Balmer BC, Wells RS, et al (2010) Eosinophilia and biotoxin exposure in bottlenose dolphins (Tursiops truncatus) from a coastal area impacted by repeated mortality events. Environ Res 110: 548–555. 10.1016/j.envres.2010.05.003 20537621

[32] Chapra SC, Boehlert B, Fant C, Bierman VJ Jr., Henderson J, et al (2017) Climate Change Impacts on Harmful Algal Blooms in U.S. Freshwaters: A Screening-Level Assessment. Environ Sci Technol 51: 8933–8943. 10.1021/acs.est.7b01498 28650153

[33] Venn-Watson SK, Jensen ED, Smith CR, Xitco M, Ridgway SH (2015) Evaluation of annual survival and mortality rates and longevity of bottlenose dolphins (Tursiops truncatus) at the United States Navy Marine Mammal Program from 2004 through 2013. J Am Vet Med Assoc 246: 893–898. 10.2460/javma.246.8.893 25835174

[34] Stolen MK, Durden WN, Odell DK (2007) Historical synthesis of bottlenose dolphin (tursiops truncatus) stranding data in the Indian River Lagoon system, Florida, from 1977–2005. Florida Scientist 70: 45–54.

[35] NOAA (2015) 2013–2015 Bottlenose Dolphin Unusual Mortality Event in the Mid-Atlantic. Health & Stranding: National Oceanic and Atmospheric Administration

[36] Clark LS, Turner JP, Cowan DF (2005) Involution of lymphoid organs in bottlenose dolphins (Tursiops truncatus) from the western Gulf of Mexico: implications for life in an aquatic environment. Anat Rec A Discov Mol Cell Evol Biol 282: 67–73. 10.1002/ar.a.20147 15622514

[37] Bonsembiante F, Centelleghe C, Rossi G, Giglio S, Madeo E, et al (2017) Clinico-pathological findings in a striped dolphin (Stenella coeruleoalba) affected by rhabdomyolysis and myoglobinuric nephrosis (capture myopathy). J Vet Med Sci 79: 1013–1018. 10.1292/jvms.17-0023 28442646 PMC5487775

[38] Pintore MD, Mignone W, Di Guardo G, Mazzariol S, Ballardini M, et al (2018) Neuropathologic Findings in Cetaceans Stranded in Italy (2002–14). J Wildl Dis 54: 295–303. 10.7589/2017-02-035 29369721

[39] Jepson PD, Deaville R, Acevedo-Whitehouse K, Barnett J, Brownlow A, et al (2013) What caused the UK's largest common dolphin (Delphinus delphis) mass stranding event? PLoS One 8: e60953 10.1371/journal.pone.0060953 23646103 PMC3640001

[40] Parsons EC, Dolman SJ, Wright AJ, Rose NA, Burns WC (2008) Navy sonar and cetaceans: just how much does the gun need to smoke before we act? Mar Pollut Bull 56: 1248–1257. 10.1016/j.marpolbul.2008.04.025 18534632

[41] Berry DL, Goleski JA, Koch F, Wall CC, Peterson BJ, et al (2015) Shifts in Cyanobacterial Strain Dominance during the Onset of Harmful Algal Blooms in Florida Bay, USA. Microb Ecol 70: 361–371. 10.1007/s00248-014-0564-5 25661475

[42] Badylak S, Phlips EJ (2004) Spatial and temporal patterns of phytoplankton composition in a subtropical coastal lagoon, the Indian River Lagoon, Florida, USA. Plankton Research 26: 1229–1247.

[43] Sneed JM, Meickle T, Engene N, Reed S, Gunasekera S, et al (2017) Bloom dynamics and chemical defenses of benthic cyanobacteria in the Indian River Lagoon, Florida. Harmful Algae 69: 75–82. 10.1016/j.hal.2017.10.002 29122244

[44] Sehein T, Richlen ML, Nagai S, Yasuike M, Nakamura Y, et al (2016) Characterization of 17 New Microsatellite Markers for the Dinoflagellate Alexandrium Fundyense (Dinophyceae), a Harmful Algal Bloom Species. J Appl Phycol 28: 1677–1681. 10.1007/s10811-015-0681-7 27274617 PMC4890638

[45] Morton S (2014) US DOC/NOAA/NOS > National Centers for Coastal Ocean Science (2014) Biological, chemical, and physical data from the Phytoplankton Monitoring Network from 13 Sep 2001 to 7 Mar 2013 (NODC Accession 0117942). Version 1.1 2014 ed: National Oceanographic Data Center, NOAA.

[46] Geraci JR, Lounsbury VJ (1993) Marine Mammals Ashore, A Field Guide for Strandings: Texas A&M University Sea Grant Publication. 344 p.

[47] Barco SG, Walton WJ, Harms CA, George RH, D’Eri LR, et al (2016) Collaborative development of recommendations for euthanasia of stranded cetaceans. NOAA Technical Memorandum NMFS-OPR-56: http://bit.ly/2ydE2CK

[48] Montalvo Villalba MC, Cruz Martinez D, Ahmad I, Rodriguez Lay LA, Bello Corredor M, et al (2017) Hepatitis E virus in bottlenose dolphins Tursiops truncatus. Dis Aquat Organ 123: 13–18. 10.3354/dao03085 28177289

[49] Mondo K, Broc Glover W, Murch SJ, Liu G, Cai Y, et al (2014) Environmental neurotoxins beta-N-methylamino-l-alanine (BMAA) and mercury in shark cartilage dietary supplements. Food Chem Toxicol 70: 26–32. 10.1016/j.fct.2014.04.015 24755394

[50] Banack SA, Cox PA (2003) Biomagnification of cycad neurotoxins in flying foxes: Implications for ALS-PDC in Guam. Neurology 61: 387–389. 12913204 10.1212/01.wnl.0000078320.18564.9f

[51] Banack SA, Johnson HE, Cheng R, Cox PA (2007) Production of the neurotoxin BMAA by a marine cyanobacterium. Mar Drugs 5: 180–196. 18463731 10.3390/md504180 PMC2365698

[52] Glover WB, Baker TC, Murch SJ, Brown PN (2015) Determination of beta-N-methylamino-L-alanine, N-(2-aminoethyl)glycine, and 2,4-diaminobutyric acid in Food Products Containing Cyanobacteria by Ultra-Performance Liquid Chromatography and Tandem Mass Spectrometry: Single-Laboratory Validation. J AOAC Int 98: 1559–1565. 10.5740/jaoacint.15-084 26651568

[53] Baker TC, Tymm FJM, Murch SJ (2018) Assessing Environmental Exposure to beta-N-Methylamino-L-Alanine (BMAA) in Complex Sample Matrices: a Comparison of the Three Most Popular LC-MS/MS Methods. Neurotox Res 33: 43–54. 10.1007/s12640-017-9764-3 28643233

[54] Mirra SS, Hart MN, Terry RD (1993) Making the diagnosis of Alzheimer’s disease. A primer for practicing pathologists. Arch Pathol Lab Med 117: 132–144. 8427562

[55] Guzman-Verri C, Gonzalez-Barrientos R, Hernandez-Mora G, Morales JA, Baquero-Calvo E, et al (2012) Brucella ceti and brucellosis in cetaceans. Front Cell Infect Microbiol 2: 3 10.3389/fcimb.2012.00003 22919595 PMC3417395

[56] Bogomolni AL, Pugliares KR, Sharp SM, Patchett K, Harry CT, et al (2010) Mortality trends of stranded marine mammals on Cape Cod and southeastern Massachusetts, USA, 2000 to 2006. Dis Aquat Organ 88: 143–155. 10.3354/dao02146 20225675

[57] Cox PA, Banack SA, Murch SJ (2003) Biomagnification of cyanobacterial neurotoxins and neurodegenerative disease among the Chamorro people of Guam. Proc Natl Acad Sci U S A 100: 13380–13383. 10.1073/pnas.2235808100 14612559 PMC263822

[58] Sarasa M, Pesini P (2009) Natural non-trasgenic animal models for research in Alzheimer’s disease. Curr Alzheimer Res 6: 171–178. 10.2174/156720509787602834 19355852 PMC2825666

[59] Gunn-Moore D, Kaidanovich-Beilin O, Gallego Iradi MC, Gunn-Moore F, Lovestone S (2017) Alzheimer’s disease in humans and other animals: A consequence of postreproductive life span and longevity rather than aging. Alzheimers Dement. 10.1016/j.jalz.2017.08.014 28972881

[60] Andrew AS, Chen CY, Caller TA, Tandan R, Henegan PL, et al (2018) Toenail mercury Levels are associated with amyotrophic lateral sclerosis risk. Muscle Nerve. 10.1002/mus.26055 PMC6034986 29314106

[61] Rush T, Liu X, Lobner D (2012) Synergistic toxicity of the environmental neurotoxins methylmercury and beta-N-methylamino-L-alanine. Neuroreport 23: 216–219. 22314682 10.1097/WNR.0b013e32834fe6d6

[62] Eiser AR (2017) Why does Finland have the highest dementia mortality rate? Environmental factors may be generalizable. Brain Res 1671: 14–17. 10.1016/j.brainres.2017.06.032 28687259

