# Supplementary material for: Cyanobacterial neurotoxin BMAA and brain pathology in stranded dolphins
Source: PLoS One. 2019 Mar 20;14(3):e0213346. doi: 10.1371/journal.pone.0213346 (PMC6426197; doi:10.1371/journal.pone.0213346)
Supplement: S1 Table — (DOCX) [file pone.0213346.s001.docx]

| **No.** | **Common Name** | **Species** | **Agency ID** | **Observed** | **Stranding Date** | | **Stranding**  **Location** | | | **Sex** | | | **Age Class** | | **Length (cm)** | | **Weight (kg)** | | **BMAA (μg/g)** | |
| --- | --- | --- | --- | --- | --- | --- | --- | --- | --- | --- | --- | --- | --- | --- | --- | --- | --- | --- | --- | --- |
|  | **Florida** |  |  |  |  | |  | | |  | | |  | |  | |  | |  | |
| 1 | Bottlenose Dolphin | *Tursiops truncatus* | Hubbs 0805 Tt | Floating | 01/30/08 | | Atlantic Ocean | | | Female | | | Adult | | 231 | | 249 | | *ND* **^Δ^** | |
| 2 | Bottlenose Dolphin | *Tursiops truncatus* | Hubbs 0720 Tt | Beached | 02/22/07 | | Atlantic Ocean | | | Female | | | Adult | | 240 | | 335 | | 114 | |
| 3 | Bottlenose Dolphin | *Tursiops truncatus* | Hubbs 0717 Tt | Beached | 02/11/07 | | Indian River | | | Male | | | Adult | | 250 | | *NA* | | 295 | |
| 4 | Bottlenose Dolphin | *Tursiops truncatus* | Hubbs 0630 Tt | Beached | 05/24/06 | | Banana River | | | Female | | | Adult | | 236 | | 305 | | 335 | |
| 5 | Bottlenose Dolphin | *Tursiops truncatus* | Hubbs 0541 Tt | Swimming | 12/26/05 | | Indian River | | | Male | | | Adult | | 245 | | 335 | | 541 | |
| 6  7  8  9  10  11  12  13  14  15  16  17  18 | Bottlenose Dolphin  Bottlenose Dolphin  **Massachusetts**  Common Dolphin  Common Dolphin  Common Dolphin  Common Dolphin  Common Dolphin  Common Dolphin  Common Dolphin  **Reference**  Positive Control  Negative Control  Alzheimer Disease  ALS | *Tursiops truncatus*  *Tursiops truncatus*  *Delphinus delphis*  *Delphinus delphis*  *Delphinus delphis*  *Delphinus delphis*  *Delphinus delphis*  *Delphinus delphis*  *Delphinus delphis*  *Homo Sapiens*  *Homo Sapiens*  *Homo Sapiens*  *Homo Sapiens* | Hubbs 0636 Tt  PCNMF S08-01  IFAW 12-228 Dd  IFAW 12-223 Dd  IFAW 12-200 Dd  IFAW 12-198 Dd  IFAW 12-229 Dd  IFAW 12-205 Dd  IFAW 12-201 Dd  UMBEB AD_ON  UMBEB UC_JP  *Pablo et al. 2009*  *Pablo et al. 2009* | Beached  Floating  Beached  Beached  Beached  Beached  Swimming  Beached  Beached | 07/11/06  02/08/08  04/05/12  03/12/12  03/07/12  03/06/12  04/03/12  03/11/12  03/09/12 | | Atlantic Ocean  Gulf of Mexico  Black Fish Creek  Skaget Beach  Corporation Beach  Power’s Landing  Black Fish Creek  Duck Creek  Skaget Beach | | | Male  Female  Male  Male  Male Female  Male Female  Female | | | Sub-Adult  Adult  Adult  Sub-Adult  Sub-Adult  Adult  Sub-Adult  Sub-Adult  Adult  Adult  Adult  Adult  Adult | | 223  253  211  185  157  186  181  167  206 | | 285  *NA*  86  *NA*  42  77  *NA*  57  103 | | 675  748  20**  111**  127  129  157**  170  320  209  *ND*  111  134 | |
|  | **Δ,** Boat Injury **, Brucellosis | ***NA***, Not Available | ***ND***, Not Detected |  |  | |  | |  | |  | | |  | |  | |  | |  |
|  |  |  |  |  |  |  | |  |  | | |  | |  | |  | |  |  |  |
|  |  |  |  |  |  |  | |  |  | | |  | |  | |  | |  |  |  |
|  |  |  |  |  |  |  | |  |  | | |  | |  | |  | |  |  |  |
|  |  |  |  |  |  |  | |  |  | | |  | |  | |  | |  |  |  |
|  |  |  |  |  |  |  | |  |  | | |  | |  | |  | |  |  |  |

**S1 Table. Stranded Dolphin Demographics**
