# Supplementary material for: Cyanobacterial neurotoxin BMAA and brain pathology in stranded dolphins
Source: PLoS One. 2019 Mar 20;14(3):e0213346. doi: 10.1371/journal.pone.0213346 (PMC6426197; doi:10.1371/journal.pone.0213346)
Supplement: S2 Table — (DOCX) [file pone.0213346.s002.docx]

| **Regions** | **Sampling**  **Dates and Measures** | | **Water Temperature**  **Mean and Range (^o^C)** | | **Air Temperature**  **Mean and Range (^o^C)** | | **Salinity (ptt)** | **Phytoplankton Species**  **No., Mean and Range (cells/L)** | | |
| --- | --- | --- | --- | --- | --- | --- | --- | --- | --- | --- |
| **Cape Cod Bay ^A^** | 2007 − 2008 | n=27 | 4.7 ± 0.6 | 2.0 – 10.0 (8.0) | 3.7 ± 0.7 | 1.0 – 14.0 (13) | 32.4 ± 0.1 | n= 14 | 76.0 ± 33.5 | 1.0 – 855.0 (854.0) |
| **Indian River ^B-E^** | 2008 − 2012 | n=519 | 23.7 ± 0.2 | 8.0 – 32.0 (24.0) | 24.5 ± 0.3 | 6.6 – 32 (25.4) | 28 ± 0.3 | n= 45 | 204.3 ± 128.7 | 1.0 – 50112.0 (50111.0) |
| **Banana River ^F^** | 2010 − 2013 | n=191 | 24.3 ± 0.3 | 6.0 – 31.0 (25.0) | 24.5 ± 0.4 | 6.0 – 31.0 (25.0) | 31. 9 ± 0.2 | n= 34 | 28.2 ± 8.9 | 1.0 – 1005.0 (1004.0) |
| **Atlantic Ocean ^G-K^** | 2008 − 2011 | n=683 | 22.7 ± 0.2 | 10.0 – 30.0 (20.0) | 23.9 ± 0.2 | 10.0 − 38.5 (28.5) | 32.8 ± 0.2 | n= 42 | 25.1 ± 2.4 | 1.0 – 740.0 (739.0) |
| **Gulf of Mexico ^L-N^** | 2008 − 2011 | n=199 | 27.6 ± 0.3 | 14.0 − 31.5 (17.5) | 27.9 ± 0.3 | 19.0 – 36.0 (17.0) | 29.9 ± 0.2 | n= 33 | 32.9 ± 5.3 | 1.0 – 572.0 (571.0) |

**S2 Table. Surveillance data from the Phytoplankton Monitoring Network obtained in regions of dolphin stranding**

***Sampling sites:* ^A^** Macmillan Pier; **^B^**CCBC Testing Platform; **^C^**192 Causeway Seawall; **^D^** Eau Gallie Pier; **^E^** Florida Oceanographic Dock; **^F^** Banana River; **^G^** St. Augustine Inlet; **^H^** Crescent Beach Park; **^I^** Sebastian Inlet; **^J^** Fort Pierce Inlet; **^K^** FIT Vero Beach Marine Lab; **^L^** Gulf Specimen Marine Lab Pier; **^M^** Sanibel Sea School (Beach); **^N^** Tampa Bay at Manatee River
