# Supplementary material for: Cyanobacterial neurotoxin BMAA and brain pathology in stranded dolphins
Source: PLoS One. 2019 Mar 20;14(3):e0213346. doi: 10.1371/journal.pone.0213346 (PMC6426197; doi:10.1371/journal.pone.0213346)
Supplement: S3 Table — (DOCX) [file pone.0213346.s003.docx]

**S3 Table. Statistical Analyses**

| **Comparison** | **Measure** | **Group** | **Sample Size** | **Test** | **One or two-tailed** | **α-Value** | **Difference In Median** | **P-Value** | **Cohen's *d*** | **R^2^** | **r** |
| --- | --- | --- | --- | --- | --- | --- | --- | --- | --- | --- | --- |
| Correlation | [BMAA] | Length vs. [BMAA] | n=13 | Pearson | one-tailed | 0.05 | *N/A* | 0.0151 | *N/A* | 0.36 | 0.60 |
| Correlation | Aβ^+^ No. | ACtx vs. VCtx | n=7 | Spearman | one-tailed | 0.05 | *N/A* | 0.0119 | *N/A* | *N/A* | 0.86 |
| Correlation | Aβ^+^ Size | ACtx vs. VCtx | n=7 | Spearman | one-tailed | 0.05 | *N/A* | 0.0833 | *N/A* | *N/A* | 0.61 |
| Correlation | [BMAA] | [BMAA] vs. ACtx Aβ^+^ No. | n=7 | Spearman | one-tailed | 0.05 | *N/A* | 0.3565 **^NS^** | *N/A* | *N/A* | 0.1786 |
| Correlation | [BMAA] | [BMAA] vs. VCtx Aβ^+^ No. | n=7 | Spearman | one-tailed | 0.05 | *N/A* | 0.1000 **^NS^** | *N/A* | *N/A* | 0.5714 |
| Correlation | [BMAA] | [BMAA] vs. ACtx Aβ^+^ Size | n=7 | Spearman | one-tailed | 0.05 | *N/A* | 0.2488 **^NS^** | *N/A* | *N/A* | 0.3214 |
| Correlation | [BMAA] | [BMAA] vs. VCtx Aβ^+^ Size | n=7 | Spearman | one-tailed | 0.05 | *N/A* | 0.4817 **^NS^** | *N/A* | *N/A* | -0.03571 |
| Multiple | [Con] | AVE [BMAA] vs. [DAB] vs. [AEG] | n=6 | ANOVA | *N/A* | 0.05 | *N/A* | 0.0001 | 2.04 | *N/A* | *N/A* |
| Single | Length | Common vs. Bottlenose | n=7 | t-Test | two-tailed | 0.05 | -55 cm | 0.0006 | 3.53 | *N/A* | *N/A* |
| Single | Weight | Common vs. Bottlenose | n=5 | t-Test | two-tailed | 0.05 | -228 kg | 0.0119 | 7.43 | *N/A* | *N/A* |
| Single | [BMAA] | Common vs. Bottlenose | n=7 vs. 6 | t-Test | two-tailed | 0.05 | -309 μg/g | 0.0350 | 1.65 | *N/A* | *N/A* |
| Single | Aβ^+^ No. | ACtx vs. VCtx | n=7 | t-Test **^(PD)^** | two-tailed | 0.05 | 9 plaques | 0.0313 | 0.61 | *N/A* | *N/A* |
| Single | Aβ^+^ Size | ACtx vs. VCtx | n=7 | t-Test | two-tailed | 0.05 | -7 pixels | 0.5781 **^NS^** | 0.18 | *N/A* | *N/A* |
| Single | Aβ^+^ No. | Adult vs. Sub-adult | n=3 vs. 4 | t-Test | one-tailed | 0.05 | -18 plaques | 0.5000 **^NS^** | 0.24 | *N/A* | *N/A* |
| Single | Aβ^+^ Size | Adult vs. Sub-adult | n=3 vs. 4 | t-Test | one-tailed | 0.05 | -12 plaques | 0.0571 **^NS^** | 1.55 | *N/A* | *N/A* |
| Single | Aβ^+^ No. | Brucellosis vs. NIF | n=3 vs. 4 | t-Test | one-tailed | 0.05 | -38 plaques | 0.0286 | 2.74 | *N/A* | *N/A* |
| Single | Aβ^+^ Size | Female vs. Male | n=3 vs. 4 | t-Test | two-tailed | 0.05 | 4 pixels | 0.8571 **^NS^** | 0.040 | *N/A* | *N/A* |
| Single | Aβ^+^ No. | Female vs. Male | n=3 vs. 4 | t-Test | two-tailed | 0.05 | 31 plaques | 0.4000 **^NS^** | 0.89 | *N/A* | *N/A* |

**Aβ^+^**: amyloid beta; **ACtx:** auditory cortex; **[Con]**: concentration; **VCtx:** visual cortex; **N/A:** not applicable; **NIF**: non-infected; **No.**: number; **NS:** not significant; **PD**: paired;

**BMAA:** β-methylamino-L-alanine
