# Supplementary material for: Cyanobacterial neurotoxin BMAA and brain pathology in stranded dolphins
Source: PLoS One. 2019 Mar 20;14(3):e0213346. doi: 10.1371/journal.pone.0213346 (PMC6426197; doi:10.1371/journal.pone.0213346)
Supplement: S4 Table — (DOCX) [file pone.0213346.s004.docx]

| **Agency ID** | **BMAA (μg/g)** | | **AEG (μg/g)** | | **DAB (μg/g)** | |  |
| --- | --- | --- | --- | --- | --- | --- | --- |
| Hubbs 0630 Tt | 0.084 ± 0.084 | | *ND* | | 1.196 ± 0.191 | |  |
| Hubbs 0805 Tt | 0.095 ± 0.056 | | *ND* | | 2.284 ± 0.262 | |  |
| Hubbs 0541 Tt | 0.198 ± 0.103 | | 0.402 ± 0.402 | | 1.180 ± 0.346 | |  |
| Hubbs 0636 Tt | 0.604 ± 0.171 | | *ND* | | 4.003 ± 0.978 | |  |
| ***ND***, Not Detected | |  | |  | |  | |

**S4 Table. LC-MS/MS detection of BMAA**
