# Supplementary material for: Cyanobacterial neurotoxin BMAA and brain pathology in stranded dolphins
Source: PLoS One. 2019 Mar 20;14(3):e0213346. doi: 10.1371/journal.pone.0213346 (PMC6426197; doi:10.1371/journal.pone.0213346)
Supplement: S5 Table — (DOCX) [file pone.0213346.s005.docx]

**S5 Table. Comparison of BMAA and Aβ^+^ plaques in dolphin brain**

| **Agency ID** | **BMAA (μg/g)** | **No. of Aβ^+^ Plaques** | **Size of Aβ^+^ Plaques (Px)** |
| --- | --- | --- | --- |
| IFAW 12-228 Dd | 20** | 45 | 70 |
| IFAW 12-223 Dd | 111** | 43 | 112 |
| IFAW 12-200 Dd | 127 | 78 | 77 |
| IFAW 12-198 Dd | 129 | 74 | 82 |
| IFAW 12-229 Dd | 157** | 40 | 90 |
| IFAW 12-205 Dd | 170 | 71 | 89 |
| IFAW 12-201 Dd | 320 | 41 | 71 |
| Mean ± SE | 140 ± 31 | 52 ± 7 | 89 ± 6 |
| Min – Max | 20 - 320 | 22 - 78 | 71 - 118 |

******, Brucellosis; **SE,** Standard Error
